# Toxicometabolomics-based cardiotoxicity evaluation of Thiazolidinedione exposure in human-derived cardiomyocytes

**DOI:** 10.1007/s11306-024-02097-z

**Published:** 2024-02-23

**Authors:** Abdullah Al Sultan, Zahra Rattray, Nicholas J. W. Rattray

**Affiliations:** 1 Strathclyde Institute of Pharmacy and Biomedical Sciences, University of Strathclyde, 161 Cathedral Street, Glasgow, G4 0RE UK; 2 Faculty of Pharmacy, Kuwait University, Safat, 13110 Kuwait; 3 Strathclyde Centre for Molecular Bioscience, University of Strathclyde, Glasgow, G4 0RE UK

**Keywords:** Thiazolidinediones, Toxicometabolomics, LC–MS, Cardiotoxicity, Amino acids, Carnitines

## Abstract

**Introduction:**

Thiazolidinediones (TZDs), represented by pioglitazone and rosiglitazone, are a class of cost-effective oral antidiabetic agents posing a marginal hypoglycaemia risk. Nevertheless, observations of heart failure have hindered the clinical use of both therapies.

**Objective:**

Since the mechanism of TZD-induced heart failure remains largely uncharacterised, this study aimed to explore the as-yet-unidentified mechanisms underpinning TZD cardiotoxicity using a toxicometabolomics approach.

**Methods:**

The present investigation included an untargeted liquid chromatography–mass spectrometry-based toxicometabolomics pipeline, followed by multivariate statistics and pathway analyses to elucidate the mechanism(s)of TZD-induced cardiotoxicity using AC16 human cardiomyocytes as a model, and to identify the prognostic features associated with such effects.

**Results:**

Acute administration of either TZD agent resulted in a significant modulation in carnitine content, reflecting potential disruption of the mitochondrial carnitine shuttle. Furthermore, perturbations were noted in purine metabolism and amino acid fingerprints, strongly conveying aberrations in cardiac energetics associated with TZD usage. Analysis of our findings also highlighted alterations in polyamine (spermine and spermidine) and amino acid (L-tyrosine and valine) metabolism, known modulators of cardiac hypertrophy, suggesting a potential link to TZD cardiotoxicity that necessitates further research. In addition, this comprehensive study identified two groupings – (i) valine and creatine, and (ii) L-tryptophan and L-methionine – that were significantly enriched in the above-mentioned mechanisms, emerging as potential fingerprint biomarkers for pioglitazone and rosiglitazone cardiotoxicity, respectively.

**Conclusion:**

These findings demonstrate the utility of toxicometabolomics in elaborating on mechanisms of drug toxicity and identifying potential biomarkers, thus encouraging its application in the toxicological sciences. (245 words)

**Supplementary Information:**

The online version contains supplementary material available at 10.1007/s11306-024-02097-z.

## Introduction

Thiazolidinediones (TZDs), represented by pioglitazone (PGZ) and rosiglitazone (ROSI) agents, are a class of oral insulin-sensitising agents used to manage type 2 diabetes mellitus, or T2DM (DeFronzo et al., 2019; Wajid et al., 2019). Moreover, TZDs are cost-effective, potent insulin sensitisers that pharmacologically mediate their action by activating the peroxisome proliferator-activated receptor-gamma (PPAR-γ) nuclear receptor (Wajid et al., 2019). Independent of their metabolic actions, TZDs have been shown to exert several pleiotropic effects involving improvements in insulin resistance, endothelial dysfunction, dyslipidaemia and vascular inflammation (Chaudhury et al., 2017; DeFronzo et al., 2019). These polyhedric effects suggest a cardiovascular protective potential that encourages the selection of TZDs in T2DM treatment in parallel with the new T2DM treatment paradigm (Association, 2023).

Simultaneous with the encouraging profile of TZDs, PGZ and ROSI are widely used globally for the initial management of T2DM. Nevertheless, a few years after their approval, international T2DM management guidelines underwent major revisions (Association, 2023; Wajid et al., 2019). The strong recommendation to drop TZDs from first-line anti-diabetic agents to second-line options was stated due to major cardiovascular concerns, particularly an increased risk of heart failure (HF) associated with their usage (Association, 2023; Chaudhury et al., 2017). While the mechanisms underpinning TZDs’ undesirable cardiotoxic action remain largely unexplained, several omics-based approaches have emerged in the toxicological sciences, providing new hope for the comprehensive elucidation of chemicals’ adverse effects (Nguyen et al., 2022).

In recent decades, toxicometabolomics has progressively been established as a powerful tool in regulatory toxicology (Olesti et al., 2021). The monitoring of the pattern of metabolic changes in response to stressors over a predefined concentration and time enables the application of toxicometabolomics in a vast number of applications, including (i) the elucidation of toxicity pathways and (ii) the tracking of the toxicokinetic and toxicodynamic data of both parent drug and biotransformation products, which can further hasten the acquisition of mechanistic knowledge (Nguyen et al., 2022; Olesti et al., 2021).

Owing to the rapid advancement in analytical technologies along with the availability of bioinformatics data modelling, subsequent integration has caused a paradigm shift in the scope and delivery of toxicity-related investigations (Li et al., 2021; Nguyen et al., 2022). These new approaches have shifted the nature of output data from observation-based outcomes to a more mechanistic and targeted analysis of any particular xenobiotic in the human system (Olesti et al., 2021). To date, extensive toxicometabolomics studies have been devoted to revealing the toxicity modes of various drugs (Cabaton et al., 2018; Li et al., 2020). Using the aforementioned approach, these studies have successively reported the discovery of toxicity biomarkers, while gaining a better understanding of the underpinnings of toxicity pathways (Cabaton et al., 2018; Li et al., 2020).

In the present study, an untargeted liquid chromatography–mass spectrometry (LC–MS)-based toxicometabolomics approach, followed by multivariate statistics, has been performed to elucidate the mechanism of TZD-induced cardiotoxicity using AC16 human cardiomyocytes. The primary aim of this study was to (i) profile the biochemical pathways perturbed in TZD-treated AC16 human cardiomyocytes and (ii) identify biomarker candidates associated with such an effect that could serve as potential therapeutic targets for TZDs’ undesirable effects.

## Methods

### Reagents and chemicals

PPARγ agonists, PGZ and ROSI, were purchased from Sigma-Aldrich. The reagents used for the LC–MS analysis consisted of high-performance liquid chromatography (HPLC)-grade acetonitrile, methanol, analytical-grade formic acid and ultrapure water and were purchased from Fisher Scientific.

### Cells and cell culture

The AC16 cell line was purchased from Sigma-Aldrich. The cells were cultured in Dulbecco’s Modified Eagle’s Medium (DMEM/F-12) supplemented with 12.5% foetal bovine serum (FBS), 1% antibiotics (streptomycin and penicillin) and 2 mM L-glutamine at 37 °C in a humidified atmosphere of 5% CO_2_ (Bourgault et al., 2011; Wei et al., 2020).

### In vitro cytotoxicity measurements

The cytotoxicity of TZDs on AC16 cells was investigated using two mitochondrial assays, each of which carries a different endpoint (MTT assay (Cat. No. V13154; Thermo Fisher): implication of mitochondrial dehydrogenases; and adenosine triphosphate (ATP) assay (Part No. G7570; Promega): measurement of oxidative phosphorylation. Details on the protocol followed for each performed assay are included in the supplementary file (Sect. 1.1.1 and 1.1.2).

### Sample preparation and metabolite extraction

To profile changes in the endogenous metabolites, AC16 cells were seeded at a density of 2 × 10^6^ cells/well in six-well plates containing 2 mL of medium per well and incubated for 24 h. Following a 24-h incubation period, the cells were washed once with phosphate-buffered saline (PBS) and supplemented with either a new phenol red-free medium alone or exposed to the half-maximal inhibitory concentration of either PGZ or ROSI (details on IC_50_ determination are included in the Supplementary Sect. 1.1). After the 24 h treatment period, the plates were placed on an ice-cold metal plate, and the AC16 cells were washed with 500 µL of ice-cold PBS. Using a pre-chilled plastic cell scraper, the cells were harvested three times with 500 µL of ice-cold methanol/water (50/50, v/v) and aliquoted in microcentrifuge tubes. Subsequently, the microcentrifuge tubes were placed in liquid nitrogen. The samples were then allowed to sit for a few seconds and vortexed for 2 min. The resultant extracts were centrifuged at 12,000 g for 15 min at 4 °C. The supernatant was then collected into new microcentrifuge tubes and evaporated using a Thermo Scientific™ Savant™ SpeedVac™ to form dried metabolite extract pellets, while the recovered sediment pellets were retained for total protein quantification using the Bradford assay. The dried metabolite pellets were reconstituted in water/0.1% formic acid at volumes normalised to the relative protein content. Eventually, the reconstituted solutions were transferred to 300µL fixed insert glass vials for LC-MS analysis. Following sample preparation, quality control (QC) and blank samples were prepared. The QC samples were prepared by mixing equal volumes of all the prepared and tested samples. The blank sample, typically used to monitor background contamination or interference acquired through sample preparation, was prepared by pooling methanol/water (50/50, v/v).

### LC-MS data acquisition and processing

Metabolite extracts of the AC16 cell biomass and corresponding culture media were randomised and subsequently analysed by high-performance liquid chromatography-electrospray ionisation quadrupole orbitrap mass spectrometry (HPLC-ESI-HRMS) using a Thermo Scientific™ Vanquish™ binary LC system coupled to a Thermo Scientific™ Orbitrap Exploris™ 240 mass spectrometer. Details of the parameters for chromatographic separation and MS detection are included in the Supplementary Sect. 1.2.1.

The acquired LC-MS data were processed using Compound Discoverer 3.2 software (Thermo Fisher). Details on LC–MS metabolomics data processing are described in the Supplementary Sect. 1.2.2.

### Bioinformatics analysis

#### Univariate and multivariate data analyses

Univariate and multivariate statistical analyses were performed using R v4.3.0 and MetaboAnalyst v6.0 (https://www.metaboanalyst.ca) webserver. Before the data analyses and through Compound Discoverer 3.2 software, the spectral data were filtered by annotation filters (i.e., a full match with the predefined databases). This was followed by data normalisation using the *MSPrep* R package (Hughes et al., 2014).

Regarding multivariate analysis, principal component analysis (PCA) and orthogonal partial least squares-discriminant analysis (OPLS-DA) were developed to inspect the clustering of biological samples and model the discriminations between the experimental groups. Furthermore, random forest analysis (RF), was performed to identify the features that had the highest discriminatory power between the two experimental groups. The number of trees in this study was set to 500. Univariate analysis, Student’s t-test, was conducted to identify differentially expressed features (DEFs) between control and TZD-treated groups. The *p* and FDR values were set at 0.05.

Through combining univariate and multivariate analyses findings, features that fit on one of the following criteria—(i) variable importance in the projection (VIP) value > 1 of the OPLS-DA model, (ii) discriminant features identified by RF and (iii) significant features extracted from univariate analysis (*p*-value ≤ 0.05)—were labelled in this study as characteristic features and hence subjected for debiased sparse partial correlation analysis (DSPC)–weighted network analysis, metabolite set enrichment analysis (MSEA) and pathway analysis.

#### DSPC network, MSEA and pathway analyses

To further explore the metabolic alteration underpinning treatment conditions, the correlation among the characteristic features was determined through DSPC weighted network analysis. In addition, MSEA and pathway analyses were performed to profile the perturbed biochemical pathways in response to TZD treatment. The hypergeometric test’s *p*-values determined the pathway impact and statistical significance of the identified metabolic pathways.

#### Selection of biomarker candidates

To identify biomarker candidates associated with the cardiotoxicity of TZDs, univariate receiver operating characteristic (ROC) curves were applied. Initially, hub feature(s) identified from the DSPC network (feature(s) with the highest degree score) that were also enriched in pathways linked with TZDs’ cardiotoxicity were defined in this study as biomarker candidates. Thereafter, ROC curves were constructed and the area under the curve (AUC) was calculated to evaluate the prognostic potential of these features.

### Statistical analysis

**Fig. 1:**
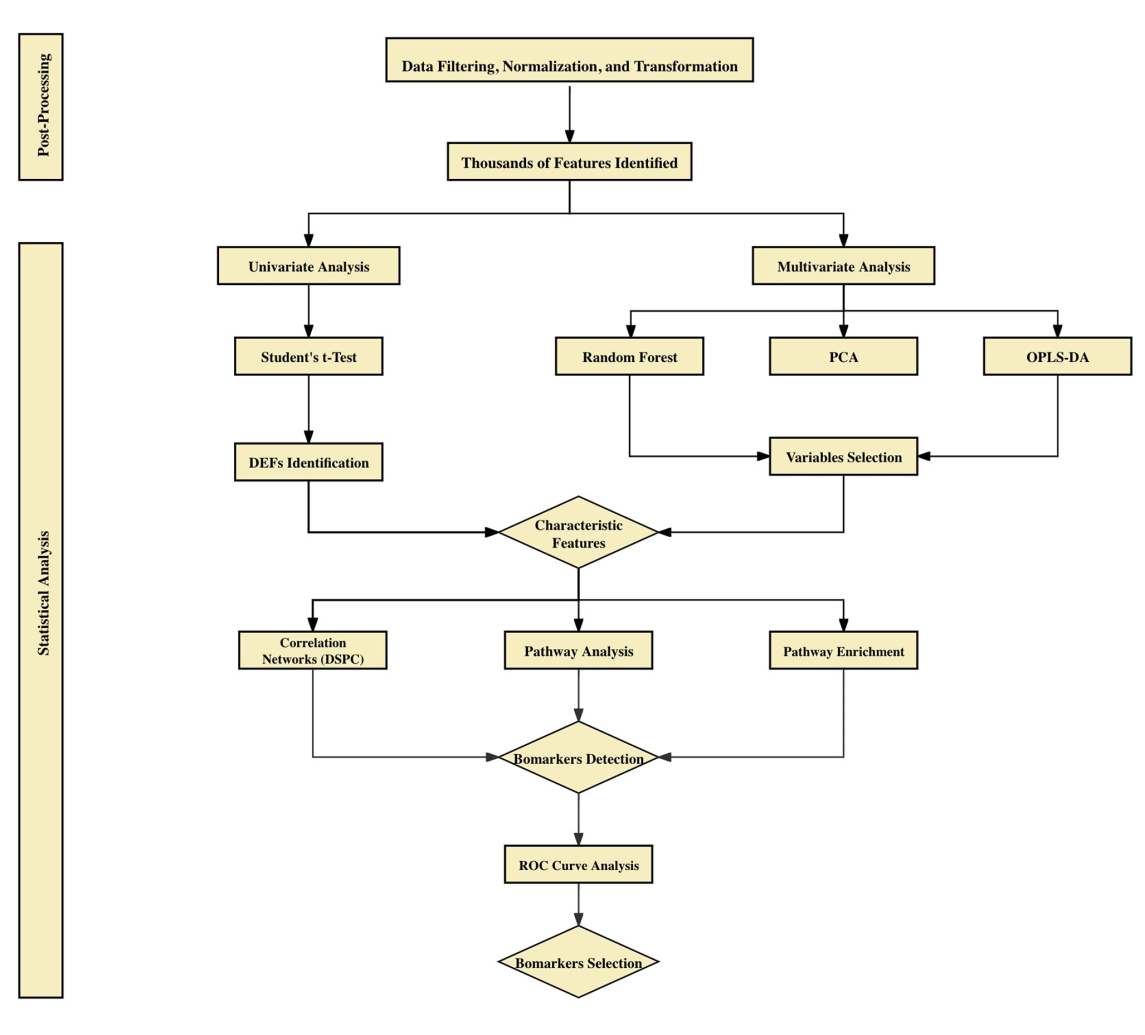
Flowchart of the toxicometabolomics pipeline applied for downstream analyses. The metabolic profiling of AC16 cells produced in response to the TZDs was characterised using an untargeted LC-MS approach. First, the raw LC-MS data were processed using Compound Discoverer, after which further data filtering and normalisation were conducted using the *MSPrep* R package. Thereafter, downstream analysis, including uni- and multi-variate analyses, was performed on the identified features. PCA was performed to identify potential outliers. Subsequently, OPLS-DA and RF analysis, both supervised techniques, were adopted as feature selectors and classifiers. Alongside the multivariate analysis, univariate analysis, Student’s t-test, was conducted to identify differentially expressed features (DEFs) between control and TZD-treated groups. The *p* and FDR values were set at 0.05. Accordingly, the characteristic features were first selected by combining the univariate and multivariate findings and then subjected to DSPC weighted network analysis, metabolite set enrichment analysis and pathway analysis. Finally, this study defined the hub features identified from the DSPC network, which were also observed to be enriched in the pathways linked to TZD’s cardiotoxicity pathogenesis, as biomarker candidates. Additionally, ROC curves were applied to evaluate the prognostic potential of the chosen candidates LC–MS: liquid chromatography–mass spectrometry; TZDs: thiazolidinediones; DEFs: differentially expressed features; PCA: principal component analysis; OPLS-DA: orthogonal partial least squares-discriminant analysis; RF: random forest; DSPC: debiased sparse partial correlation; KEGG: Kyoto Encyclopaedia of Genes and Genomes; ROC: receiver operating characteristic

Statistical analysis was conducted using R v4.3.0. Three independent experiments were performed, each conducted in triplicate (biological replicates), yielding nine samples per group. Statistical significance was determined using Student’s or Welch’s t-tests when comparing the two groups. A non-repeated one-way analysis of variance (ANOVA), followed by Dunnett’s post hoc test, was used for multiple comparisons. The correlation coefficient was assessed using Pearson and distance correlation analyses. A *p-*value ≤ 0.05 was considered statistically significant. All the downstream analyses were performed using MetaboAnalyst, otherwise delegated to R. A schematic flowchart of the toxicometabolomics pipeline applied for downstream analyses is illustrated in Fig. 1.

## Results

### In vitro characterisation of TZD cytotoxicity against AC16

The effects of PGZ and ROSI on cell viability were assessed using the MTT assay. AC16 cells were treated with a wide range of concentrations of either TZD agents (0.01, 0.1, 0.5, 1, 5, 10, and 20 µM), and the cytotoxic effect was measured after a 24-h incubation period. As shown in Figures S1a and S1b, exposure to either TZD resulted in a concentration-dependent decrease in cell viability, and the IC_50_ value against AC16 cells, calculated using the Hill equation (Goutelle et al., 2008), was found to be 4.74 µM (R^2^ = 0.9969; 95% CI 3.842–5.894) and 2.05 µM (R^2^ = 0.9816; 95% CI 1.270–3.495) in PGZ and ROSI, respectively.

Furthermore, the effect of TZD on mitochondrial ATP production was investigated using the CellTiter-Glo® assay. The administration of PGZ or ROSI at a concentration range of (1, 5, 10, 50 and 100 µM), as presented in Figures S2a and S2b, markedly depleted mitochondrial ATP production, notably in a concentration-dependent manner.

### Overview of cellular metabolome profiling under TZD treatment

To unveil TZDs’ cardiotoxic mode of action and delineate the perturbation of the cellular metabolome in response to their exposure, a toxicometabolomics approach featuring untargeted LC–MS followed by computational bioinformatics analyses was introduced, as depicted in Fig. 1.

**Fig. 2:**
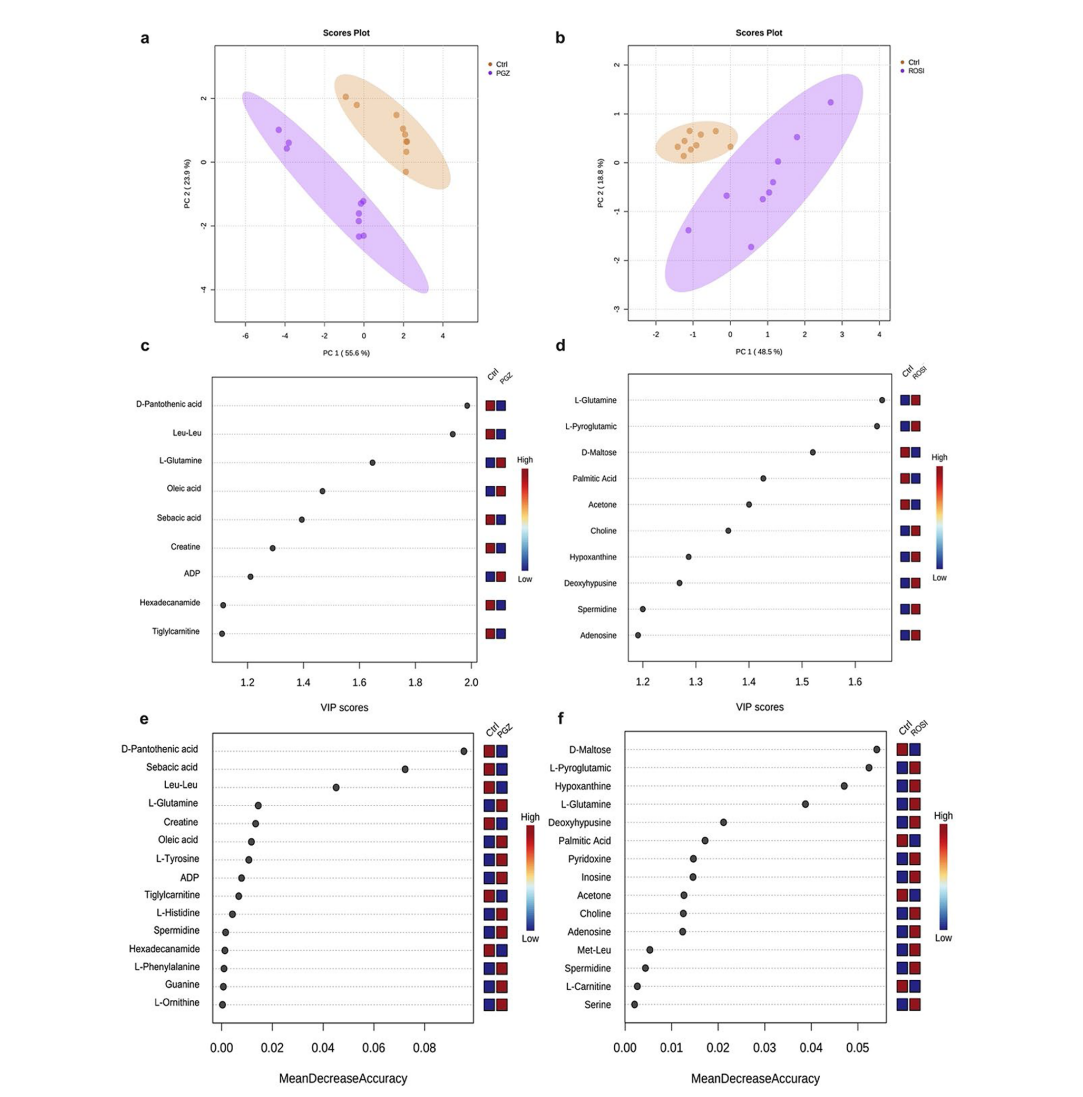
Multivariate analysis of the metabolomics data. (**a**) and (**b**) represent the 2D PCA scores plots comparing the LC–MS metabolic profiles of the PGZ-treated and ROSI-treated samples relative to the control group, respectively. Both were treated at measured IC_50_ values of 4.74µM and 2.05 µM in pioglitazone and rosiglitazone, respectively. In both PCA plots, the shaded circles represent 95% confidence intervals, while the coloured dots denote the individual samples. (**c**) and (**d**) illustrate the VIP score plots of the 10 most influential features responsible for the separation noted between the PGZ-treated vs. control groups and the ROSI-treated vs. control groups in the OPLS-DA model, respectively. Furthermore, (**e**) and (**f**) denote the random forest analysis, showing the discriminant features with the highest discriminatory power between the treated and control groups (PGZ in (**e**) and ROSI in (**f**)). In both the random forest and VIP plots, the colour code indicates higher (red) or lower (blue) concentrations PGZ: pioglitazone; ROSI: rosiglitazone; LC–MS: liquid chromatography–mass spectrometry; PCA: principal component analysis; OPLS-DA: orthogonal partial least squares-discriminant analysis; VIP: variable importance in projection

Univariate and multivariate statistical analyses were performed to decipher the metabolic perturbation in AC16 cells following TZD exposure. An unsupervised two-component PCA plot was constructed to delineate the overall similarities and heterogeneity in the clustering of the biological samples. In both experiments, the PCA scores plots indicated marked separation among the sampling data, showing a distinct metabolic profile that was yielded after TZD exposure (Fig. 2a and b). This distinct metabolic profile observed by PCA plots was also confirmed following the application of the OPLS-DA supervised model illustrated in Figure S3. Furthermore, the Variable Importance in Projection (VIP) measure was adopted to fingerprint the important features responsible for clustering separation. With respect to PGZ treatment, 9 features were found with VIP scores > 1, as listed in Fig. 2c. In contrast, several influential features were extracted from the later model in response to ROSI exposure (Fig. 2d), including amino acid-related products (e.g. L-glutamine), purines and purine derivatives (hypoxanthine), polyamines (spermidine), inosine and others. Furthermore, the putative features were ranked using the mean decrease accuracy measure integrated into the RF analysis. Regarding the PGZ experiment, the RF classification, as shown in Figure S4a, demonstrated an outstanding prediction of the treated group; nevertheless, the classification exhibited less accuracy in the control group, with a 0.0556 out-of-bag (OOB) error rate. The RF variable importance plot identified a number of discriminant features important in classifying the data, including amino acid products (e.g., L-tyrosine, valine), creatine and mitochondrial-derived metabolites such as triglylcarnitine (Fig. 2e). However, the RF classification model extracted from ROSI data, as illustrated in Figure S4b, predicted the control excellently, while the prediction of the treated class was less accurate, with a 0.056 OOB rate. The features identified by RF that had the most influence on data classification are listed in Fig. 2f. In an attempt to further identify the DEFs, univariate analysis, Student’s t-test, was conducted, yielding 16 and 53 DEFs in response to PGZ and ROSI exposure, respectively.

Thereafter, a combination of multivariate and univariate analyses was performed to define PGZ’s and ROSI’s characteristic features. The combination analysis resulted in 27 and 63 characteristic features extracted from the PGZ and ROSI datasets, respectively, discriminating the experimental groups.

The relative distribution of these defined characteristic features across TZD-treated and control groups was measured by calculating the z-score using the following formula (Wei et al., 2012):

$${\rm{z = }}\left( {{\rm{x - \mu }}} \right){\rm{/\sigma }}$$

where x indicates sample abundance; µ represents average and σ denotes the standard deviation.

**Fig. 3:**
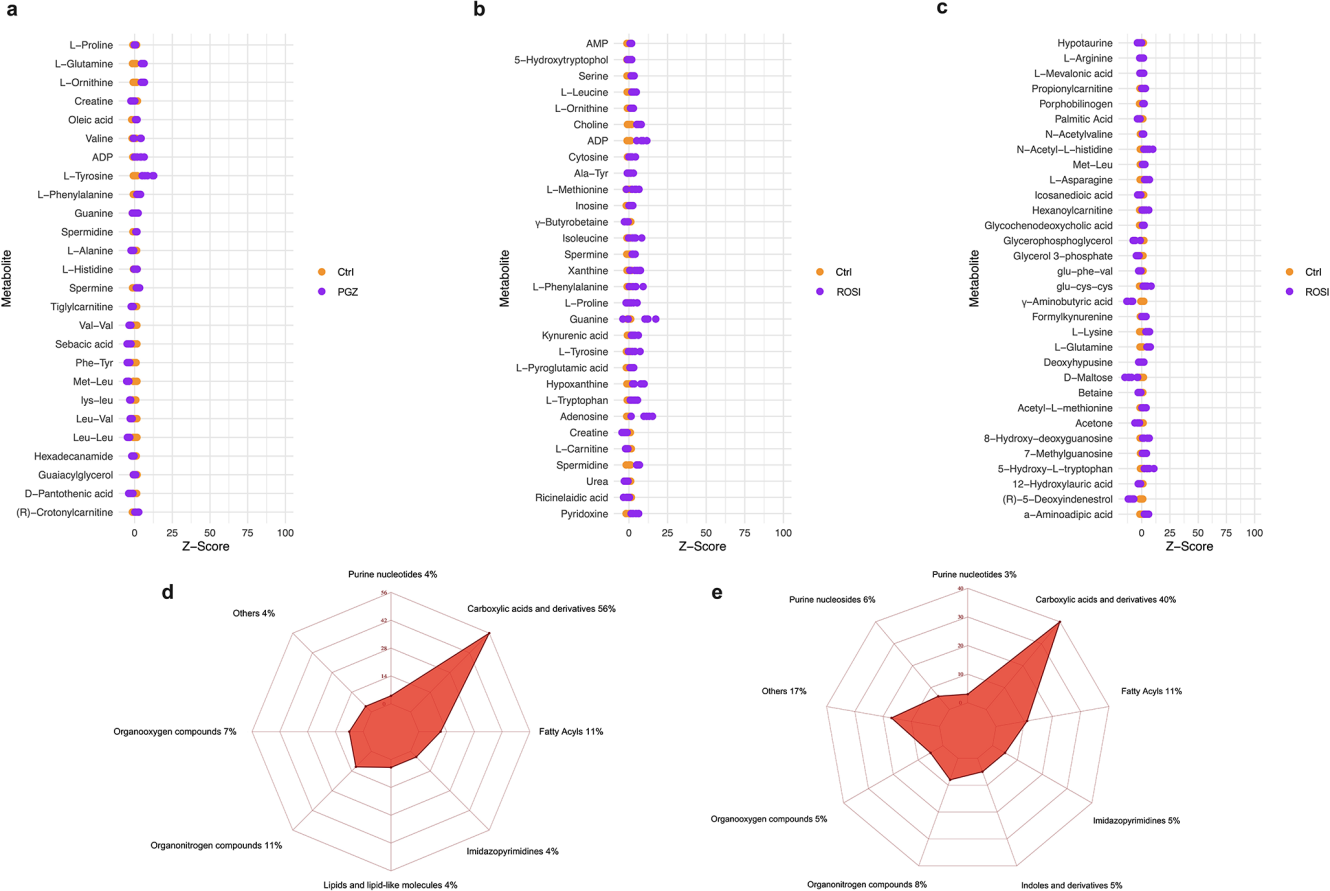
z-score plot of the characteristic features and their chemical classification. (**a**) and (**b** and **c**) present z-score plots of the characteristic features altered in the PGZ-treated and ROSI-treated samples relative to the mean in the control cells, respectively. Each point represents one metabolite in one sample, coloured according to the sample grouping. (**d**) and (**e**) show the chemical classification of the characteristic features identified from the PGZ and ROSI datasets, respectively PGZ: pioglitazone; ROSI: rosiglitazone

The z-score plot of the 27 features in the PGZ-treated group relative to the control group, as presented in Fig. 3a, exhibited metabolic perturbation in the treated group, with a z-score range of − 6 to 14 compared to the control group (z-score range: −2 to 2). The relative distribution of the 63 features altered following ROSI exposure showed z-score ranges of (− 15 to 20) and (− 2 to 2) in the treated and control groups, respectively (Fig. 3b and c). The chemical taxonomy classification of the characteristic features of each TZD agent is described in Fig. 3d and e.

### DSPC algorithm and correlation network construction

**Fig. 4:**
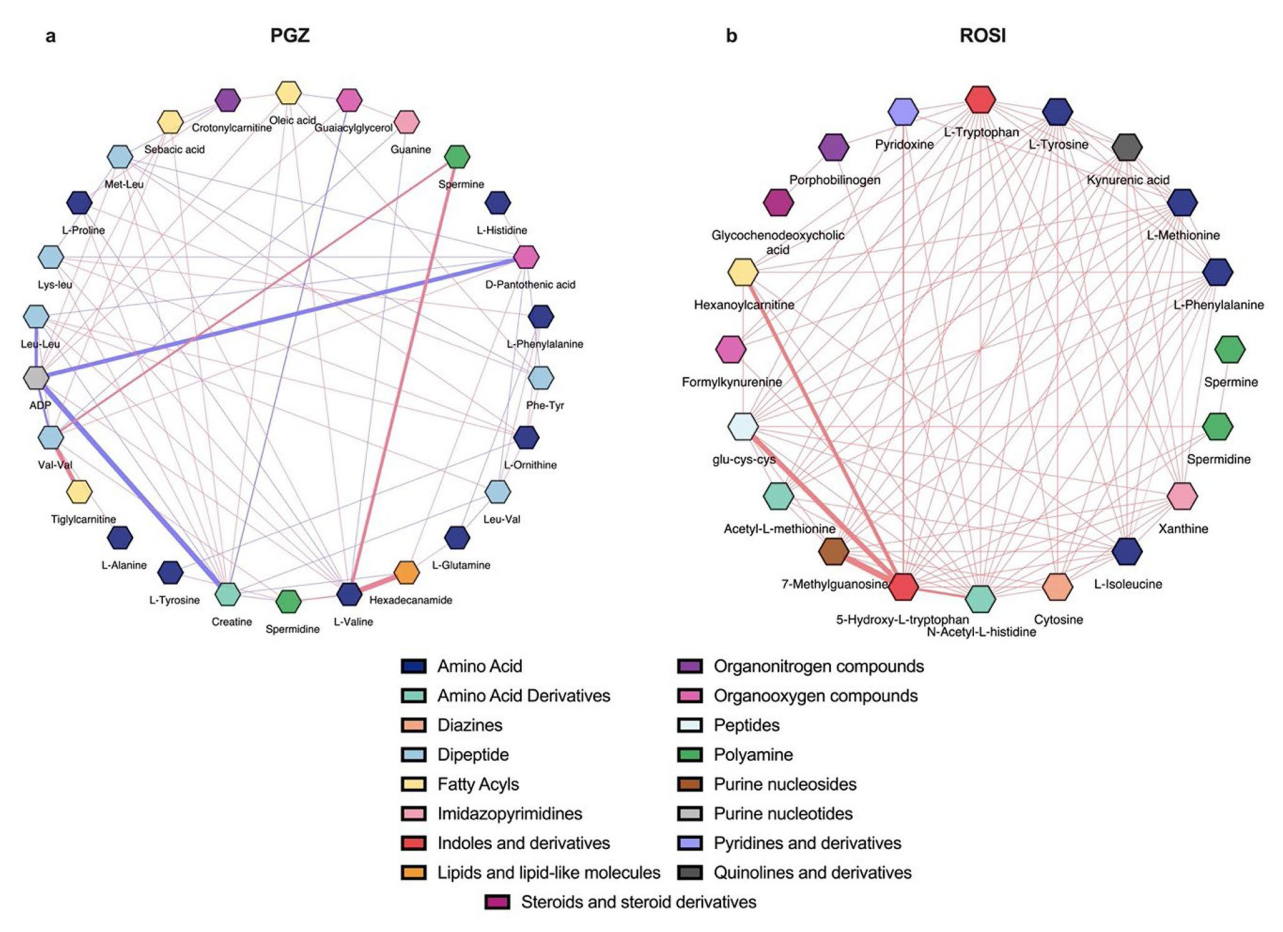
DSPC correlation network using characteristic features. (**a**) and (**b**) denote the DSPC network using PGZ’s and ROSI’s characteristic features, respectively. In both networks, the nodes represent metabolites, the red lines indicate a direct positive correlation between features, and the blue lines signify an inverse correlation. The thickness of the lines donates significance. The DSPC network analysis was performed on the basis of the graphical lasso modelling procedure, with the significance cutoff for correlation (*p*-value) set to 0.01. The range specified for the correlation coefficients was from − 1 to 1. The constructed networks were exported to the Cytoscape software platform (Cytoscape; https://cytoscape.org; v3.10.1) for visualisation DSPC: debiased sparse partial correlation; PGZ: pioglitazone; ROSI: rosiglitazone

Debiased sparse partial correlation (DSPC) was applied to explore the connectivity among PGZ’s and ROSI’s characteristic features. The PGZ-constructed network, as illustrated in Fig. 4a, revealed dense interactions among amino acids, amino acids with purine ribonucleotide (ADP) and amino acids with both polyamines (spermine and spermidine). In addition to the identified positive correlations, negative interactions were also noted, including valine with L-histidine, L-phenylalanine with guanine, and creatine with spermidine. Valine and creatine represented the main hubs with the highest degree score in the PGZ network.

Conversely, ROSI’s DSPC network, as shown in Fig. 4b, revealed dense interactions among amino acids and their derivatives, similar to PGZ. Furthermore, kynurenic acid, which is a vital bioproduct of tryptophan’s catabolism, has demonstrated strong interactions with amino acid derivatives (i.e., acetyl-L-methionine) and purine nucleosides (methylguanosine). The main hubs represented in the ROSI network include L-tryptophan and L-methionine.

### MSEA and pathway analysis

**Fig. 5:**
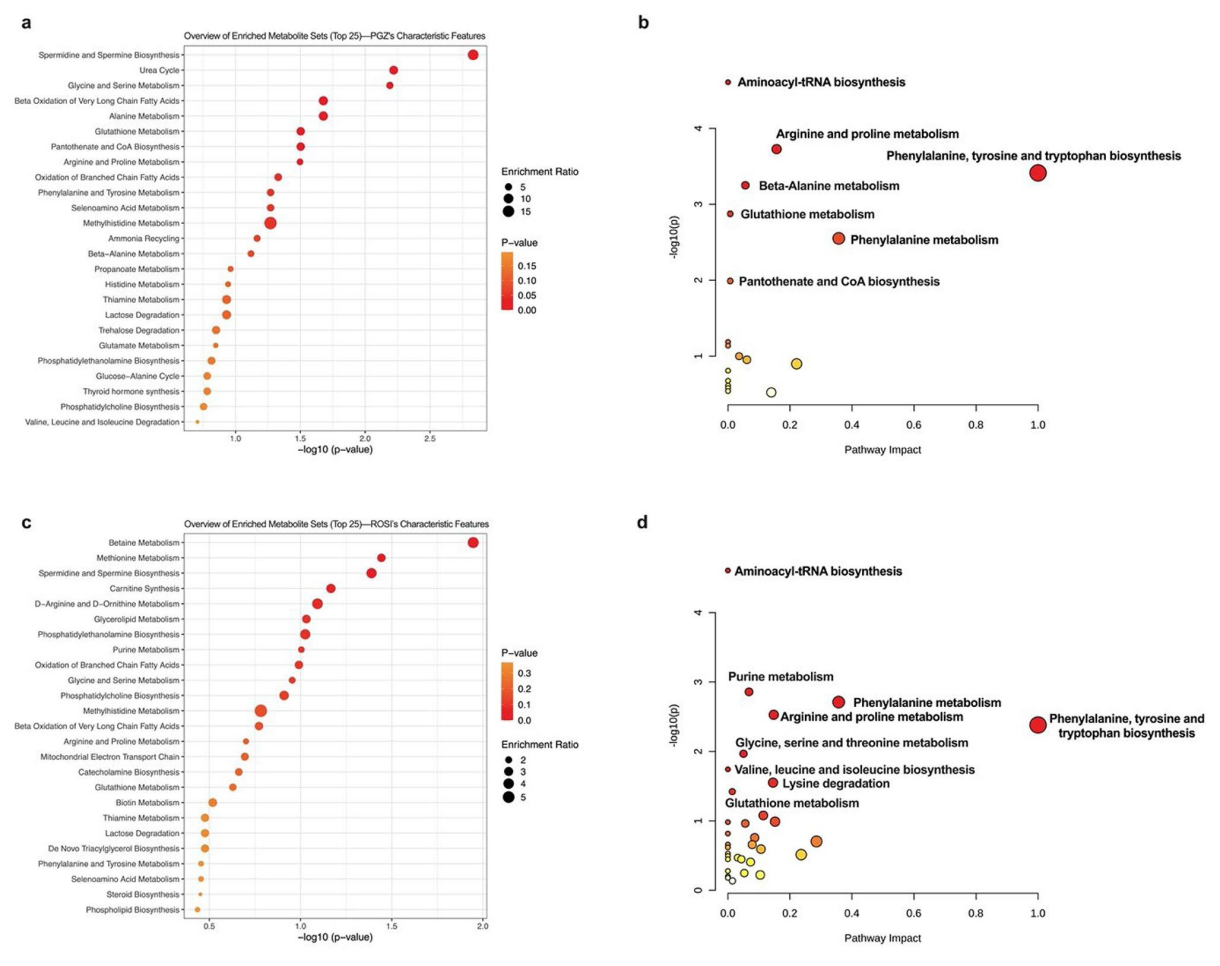
The MSEA and metabolic pathways of the characteristic features. The top 25 enriched pathways of (**a**) PGZ’s and (**c**) ROSI’s characteristic features. (**b**) and (**d**) denote the pathway analysis of the characteristic features identified from the PGZ and ROSI datasets, respectively. The size and colour of each circle in (**a**) and (**c**) reflect the enrichment ratio and significance, respectively, while those in (**b**) and (**d**) represent the pathway impact value and the *p*-value, respectively PGZ: pioglitazone; ROSI: rosiglitazone; MSEA: metabolite set enrichment analysis

To profile the biochemical pathways perturbed in PGZ- and ROSI-treated AC16 cells, MSEA and pathway analyses were performed by mapping the drug’s characteristic features against the Kyoto Encyclopedia of Genes and Genomes (KEGG) using the MetaboAnalyst webserver. The MSEA analysis revealed that the PGZ’s characteristic features were significantly enriched in pathways linked to amino acid metabolism, energy metabolism, polyamine biosynthesis, metabolism of cofactors and others as listed in Fig. 5a. The pathway analysis results, on the other hand, showed that the highest number of metabolites were products of various amino acid metabolism and amino acid and cofactor biosynthesis (Fig. 5b).

Regarding ROSI, the MSEA, as shown in Fig. 5c, revealed that the characteristic features were significantly enriched in pathways belonging to amino acid (i.e., methionine metabolism), polyamines (spermidine and spermine biosynthesis) and betaine metabolism. The pathway-topology analysis showed a significant association between the characteristic features and pathways linked to purine metabolism, amino acid metabolism and amino acid biosynthesis, as illustrated in Fig. 5d.

### Identification and validation of biomarker candidates for TZDs’ cardiotoxicity

**Fig. 6:**
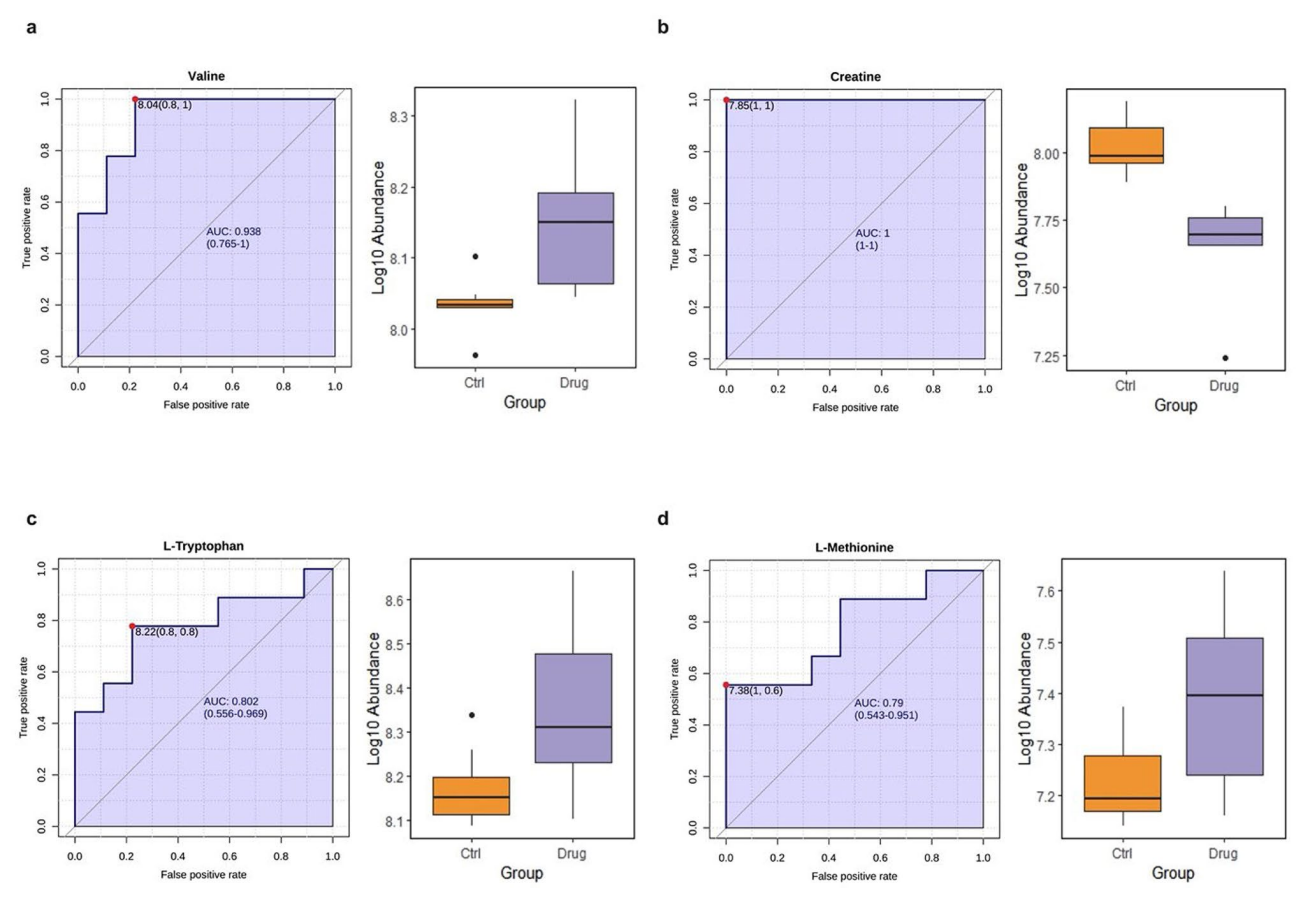
Receiver operating characteristic curves and box-plot representation for the hub features of the TZDs. (**a**) and (**b**) illustrate the receiver operating characteristic curves, along with the corresponding AUC and considering 95% confidence intervals, for PGZ’s chosen biomarkers, while (**c**) and (**d**) indicate the receiver operating characteristic analysis findings for ROSI’s biomarker candidates TZD: thiazolidinedione; PGZ: pioglitazone; ROSI: rosiglitazone; AUC: area under the curve

The hub features identified through PGZ’s and ROSI’s DSPC networks that were also enriched in pathways linked with TZDs’ cardiotoxicity were subjected to ROC analysis to evaluate their prognostic potential (Fig. 6). The ROC findings revealed excellent biomarker prediction for PGZ’s hub features; these results included valine with an AUC value of 0.938 (*p* < 0.05), as well as creatine with AUC value of 1 and *p* < 0.05. Regarding ROSI, the ROC curves had an AUC value of 0.802 (*p* < 0.05) and 0.778 (*p* < 0.05) for both L-tryptophan and L-methionine, reflecting a satisfactory overall score performance.

## Discussion

Toxicometabolomics tools have been successfully and widely employed in toxicological studies to reveal novel biochemical features and molecular biomarkers underpinning the mode of toxicity of various drugs as evident by (Cabaton et al., 2018; Dahabiyeh et al., 2020; Geng et al., 2020). Nevertheless, toxicometabolomics studies have yet to address the toxic effects associated with TZD usage (e.g., cardiotoxicity). Thus, this study was designed to employ an untargeted, LC-MS-based toxicometabolomics pipeline for comprehensive metabolic profiling of the AC16 cellular metabolome in response to the acute exposure of TZDs as a means to elucidate the uncharacterised patho-mechanistic basis of TZDs’ cardiotoxicity.

### Interpretation of results

The heterogeneity and similarity between the metabolic fingerprints of the drug-treated and control groups were assessed using multivariate statistical analyses. PCA was initially performed to inspect the clustering of the biological samples and determine potential outliers. The PCA model identified group separation, as illustrated in Fig. 2a and b. Thereafter, the supervised methods OPLS-DA and RF analysis were carried out as feature identifiers and classifiers. By combining the univariate and multivariate analysis findings, the characteristic features of each experiment were identified. In both experiments, these features predominantly include modulation in amino acids (e.g., glutamine, glycine, valine and asparagine); energy metabolites, including glutamate; and lipid content, including prenol lipids, glycerophospholipids and glycerolipids. The common and unique characteristic features, MSEA and pathway findings isolated from each experiment are illustrated via an UpSet plot in Figure S5. The modulation in characteristic features expression following TZD treatment suggests perturbation in the following major biological processes: cardiac energy metabolism and cardiac hypertrophy.

#### TZDs and cardiac energetics

It is well acknowledged that a cardiac energy deficit is a hallmark characteristic of HF. The contractile and mechanical properties of the myocardium demand a substantial, steady energy supply; hence, any disruption in the energy metabolic pathways results in drastic reduction in efficient cardiac function. Our toxicometabolomics analysis revealed modulation in the carnitine pool, including L-carnitine and triglylcarnitine, which are crucially integrated in mitochondrial fatty acid oxidation. The carnitine pool represents mitochondrial-derived metabolites primarily responsible for importing long-chain fatty acids into the mitochondria for subsequent beta-oxidation, providing roughly 70–90% of cardiac adenosine triphosphate (ATP), a process referred to as the carnitine shuttle (McCann et al., 2021). The analysis findings showed a decrease in the carnitine pool associated with TZD treatment. Of importance, enrichment in beta oxidation of very long-chain fatty acids was noted with MSEA findings, reinforcing the potential of cardiac energy failure associated with TZD administration secondary to disruption in the carnitine shuttle system and a decrease in substrate oxidation. To date, a cumulative amount of evidence has linked disruption in the carnitine profile with HF pathogenesis in both human and rodent models (Schenkl et al., 2023). Furthermore, our analysis findings revealed an increase in D-glucose levels, which could be interpreted as a compensatory mechanism to meet the energy demand in response to the disruption of fatty acid oxidation.

In the same context, our analysis revealed alterations in purine metabolites, including inosine, hypoxanthine, adenosine, and adenosine monophosphate/diphosphate (AMP/ADP), suggesting modulation in purine biosynthesis/catabolism pathways accompanying TZD treatment. It is well established that purine nucleotides play crucial roles in the synthesis of the genetic material and the energy currency of the cells, ATP (Lane & Fan, 2015). The cross-linking between the modulation in purine metabolites and TZD treatment is explained through the need to compensate for the shortage of cellular ATP. The elevated levels of both inosine and hypoxanthine suggest upregulation of the purine salvage pathway, which is a process of synthesizing purine nucleotides from nucleosides recovered from RNA and DNA degradation as a response to mitigate cardiac energy failure and increase the energy supply (Johnson et al., 2019). Nevertheless, the purine catabolism end product, hypoxanthine, has been reported to induce reactive oxygen species (ROS) generation, elevate serum cholesterol levels and worsen the progression of cardiotoxicity (Ryu et al., 2016). Therefore, the unbalancing between purine salvage and catabolism noted in our analysis could have catastrophic consequences for cardiac tissue, which necessitates further investigation.

Reflecting on the amino acid profile, modulation in branched-chain amino acids (BCAAs) represented with high levels of L-leucine, L-isoleucine and valine was noted in our analysis. Growing clinical and preclinical evidence has proposed elevated levels of BCAAs as a predictor of a wide range of cardiovascular diseases, including HF (Xiong et al., 2022). These findings surprisingly contradict the crucial roles that BCAAs play in cardiac energy metabolism. It is well recognised that BCAA oxidation acts as another fuel supply in the heart. Therefore, the high levels of BCAAs noted, and through various clinical studies performed on patients with overt cardiovascular diseases, could potentially be interpreted as a cardioprotective mechanism to promote cardiomyocyte survival. Nevertheless, the reported outcomes are inconsistent with the above-mentioned predictions. High levels of BCAAs have been shown to worsen the progression of cardiotoxicity for the following proposed reasons: (i) The contribution of BRAAs to cardiac ATP is marginal, accounting for approximately 2% of the total cardiac energetics. Therefore, elevated levels of these amino acids are not adequate for overcoming the shortage in cardiac ATP levels (Karwi & Lopaschuk, 2023). (ii) On account of recent in vivo cardiovascular studies, downregulations in key enzymes involved in BCAA oxidation have been reported, resulting in impairment in energy supply, contractile dysfunction and further accumulation of BCAAs in the myocardium (Lai et al., 2014; Sun et al., 2016). (iii) Elevated levels of BCAAs have been reported to induce mitochondrial dysfunction through mechanisms involving interfering with the electron transport chain and hence oxidative phosphorylation and altering mitochondria biogenesis through activating eNOS/NO/SIRT1 pathways (Ye et al., 2020).

#### TZDs and cardiac hypertrophy

Cardiac hypertrophy is an adaptive response prompted by physiological and pathological stressors. However, sustained hypertrophy causes a myriad of negative consequences, including the progression to HF. In our analysis, the modulation of a number of putative features that are evidently associated with cardiac hypertrophy was identified. For instance, elevated levels of polyamines, spermine and spermidine, noted in our analysis, have been linked through numerous in vivo models with cardiac hypertrophy (Giordano et al., 2010; Meana et al., 2016). Several mechanisms have been postulated to explain the cross-link association, one of which is attributed to the intrinsic ability of polyamines to modulate β-adrenoceptor signalling pathways and therefore cardiac remodelling (Giordano et al., 2010). In addition, modulation of amino acids has been associated with cardiac remodelling (Geng et al., 2020; Karwi & Lopaschuk, 2023). The high levels of BCAAs found in our toxicometabolomics analysis have been reported to activate the mammalian target of the rapamycin (mTOR) signalling pathway, a crucial hypertrophic signalling pathway implicated in HF patho-mechanisms (Xiong et al., 2022). L-tyrosine is another amino acid that has been hooked with cardiac hypertrophy, as its involvement was supported by a recent study performed to investigate the pathophysiological process of doxorubicin-induced cardiotoxicity (Geng et al., 2020). Furthermore, low levels of the nonproteinogenic amino acid γ-aminobutyric acid (GABA) were detected with TZDs. GABA is well recognized as a major inhibitory neurotransmitter with vital biological roles that are not restricted to the central nervous system but also function in peripheral tissues (Rashmi et al., 2018). In spontaneously hypertensive rats, the oral administration of GABA led to a reduction in cardiac hypertrophy (Lin et al., 2012). Hence, the low levels of GABA found in our analysis could be secondary to TZD-induced modulation of amino acid metabolism, an additional contributor factor involved in TZD cardiotoxicity. While the above-mentioned findings provide a valuable starting point, additional experiments are crucial to validate the cross-link between TZDs and cardiac hypertrophy and unravel the specific molecular pathways involved.

### Limitations and future directions

When all the results are taken together, some limitations should be addressed before drawing conclusions. Initially, in accordance with the 3Rs principle of animal experimentation, the transition in toxicological research is evolving towards animal-free in vitro and *in silico* approaches (Yu et al., 2020). This also explains the rationale behind selecting AC16 cells for our analysis. Furthermore, the well-defined cardiac signaling pathways and responsiveness to stimuli in AC16 cells, combined with their ease of culture, rapid growth, and relative cost-effectiveness compared to other models, justified their selection for our research, allowing us to effectively investigate the effects of TZDs on cardiomyocyte metabolism. While AC16 cells offer advantages for studying cardiac function, their inherent limitations, including glycolysis-dependence and fibroblast-like morphology, coupled with their dedifferentiation potential and challenges in maintaining differentiated cultures, necessitated our focus on proliferative cells for this investigation. Also, the validity of in vitro models in accurately estimating the biological complexity of the human body is still lacking (Graudejus et al., 2018; Yu et al., 2020). Moreover, when investigating the metabolic activity of cells, an in vitro model could be a limitation due to ADME effects and its limited metabolic activity compared to in vivo systems (Graudejus et al., 2018; Yu et al., 2020).

In conclusion, the present study is the first to profile the broad-scale metabolic perturbations of human AC16 induced by the TZD class of medications. The comprehensive toxicometabolomics approach employed herein has unveiled modulations in the carnitine shuttle, purine metabolism and amino acid fingerprint, each of which strongly indicate aberration in cardiac energetics associated with TZD usage. Our analysis has also pinpointed changes in polyamines and BCAA levels that are evidently associated with phenotypic alterations of cardiac tissues (hypertrophy), which indeed represents another hallmark characteristic of cardiotoxicity and a potential mechanism implicated in it. This comprehensive study also suggests the following two groupings – (i) valine and creatine, and (ii) L-tryptophan and L-methionine – which were significantly enriched in the above-mentioned mechanisms, as potential fingerprint biomarkers for PGZ and ROSI cardiotoxicity, respectively. Collectively, the results of this study suggest the LC–MS toxicometabolomics approach as a powerful platform for exploring chemical-induced perturbation in downstream molecular phenotypes, in turn pointing out a promising route for designing therapeutic targets capable of tackling these chemicals’ adverse effects.

### Electronic supplementary material

Below is the link to the electronic supplementary material.

Supplementary Material 1

## Data Availability

All mass spectrometry metabolomics data has been uploaded on to the MetaboLights database and can be found under the MTBLS9279 Study Identifier.

## References

[CR1] Association AD (2023). Standards of care in diabetes—2023 abridged for primary care providers. Clinical Diabetes.

[CR2] Bourgault S, Choi S, Buxbaum JN, Kelly JW, Price JL, Reixach N (2011). Mechanisms of transthyretin cardiomyocyte toxicity inhibition by resveratrol analogs. Biochemical and Biophysical Research Communications.

[CR3] Cabaton NJ, Poupin N, Canlet C, Tremblay-Franco M, Audebert M, Cravedi JP, Riu A, Jourdan F, Zalko D (2018). An untargeted metabolomics approach to investigate the metabolic modulations of HepG2 cells exposed to low doses of bisphenol A and 17β-estradiol. Frontiers in Endocrinology.

[CR4] Chaudhury A, Duvoor C, Reddy Dendi VS, Kraleti S, Chada A, Ravilla R, Marco A, Shekhawat NS, Montales MT, Kuriakose K, Sasapu A, Beebe A, Patil N, Musham CK, Lohani GP, Mirza W (2017). Clinical review of antidiabetic drugs: Implications for type 2 diabetes Mellitus Management. Front Endocrinol (Lausanne).

[CR5] Dahabiyeh LA, Malkawi AK, Wang X, Colak D, Mujamammi AH, Sabi EM, Li L, Dasouki M, Abdel Rahman AM (2020). Dexamethasone-induced perturbations in tissue metabolomics revealed by chemical isotope labeling LC-MS analysis. Metabolites.

[CR6] DeFronzo RA, Inzucchi S, Abdul-Ghani M, Nissen SE (2019). Pioglitazone: The forgotten, cost-effective cardioprotective drug for type 2 diabetes. Diabetes and Vascular Disease Research.

[CR7] Geng C, Cui C, Wang C, Lu S, Zhang M, Chen D, Jiang P (2020). Systematic evaluations of doxorubicin-induced toxicity in rats based on metabolomics. ACS Omega.

[CR8] Giordano, E., Flamigni, F., Guarnieri, C., Muscari, C., Pignatti, C., Stefanelli, C., Tantini, B., & Caldarera, C. M. (2010). Polyamines in cardiac physiology and disease. *Open Heart Failure Journal*, 3(1).

[CR9] Graudejus, O., Ponce Wong, R., Varghese, N., Wagner, S., & Morrison, B. (2018). Bridging the gap between in vivo and in vitro research: Reproducing in vitro the mechanical and electrical environment of cells in vivo. *Frontiers in Cellular Neuroscience*, 12.

[CR10] Hughes G, Cruickshank-Quinn C, Reisdorph R, Lutz S, Petrache I, Reisdorph N, Bowler R, Kechris K (2014). MSPrep—Summarization, normalization and diagnostics for processing of mass spectrometry–based metabolomic data. Bioinformatics.

[CR11] Johnson TA, Jinnah H, Kamatani N (2019). Shortage of cellular ATP as a cause of diseases and strategies to enhance ATP. Frontiers in Pharmacology.

[CR12] Karwi QG, Lopaschuk GD (2023). Branched-chain amino acid metabolism in the failing heart. Cardiovascular Drugs and Therapy.

[CR13] Lai L, Leone TC, Keller MP, Martin OJ, Broman AT, Nigro J, Kapoor K, Koves TR, Stevens R, Ilkayeva OR (2014). Energy metabolic reprogramming in the hypertrophied and early stage failing heart: A multisystems approach. Circulation: Heart Failure.

[CR14] Lane AN, Fan TW (2015). Regulation of mammalian nucleotide metabolism and biosynthesis. Nucleic Acids Research.

[CR16] Li YY, Ghanbari R, Pathmasiri W, McRitchie S, Poustchi H, Shayanrad A, Roshandel G, Etemadi A, Pollock JD, Malekzadeh R (2020). Untargeted metabolomics: Biochemical perturbations in Golestan Cohort Study opium users inform intervention strategies. Frontiers in Nutrition.

[CR15] Li Y, Ma L, Wu D, Chen G (2021). Advances in bulk and single-cell multi-omics approaches for systems biology and precision medicine. Briefings in Bioinformatics.

[CR17] Lin PP, Hsieh YM, Kuo WW, Lin CC, Tsai FJ, Tsai CH, Huang CY, Tsai CC (2012). Inhibition of cardiac hypertrophy by probiotic-fermented purple sweet potato yogurt in spontaneously hypertensive rat hearts. International Journal of Molecular Medicine.

[CR18] McCann MR, De la Rosa G, Rosania MV, Stringer KA (2021). L-carnitine and acylcarnitines: Mitochondrial biomarkers for precision medicine. Metabolites.

[CR19] Meana C, Rubin JM, Bordallo C, Suarez L, Bordallo J, Sanchez M (2016). Correlation between endogenous polyamines in human cardiac tissues and clinical parameters in patients with heart failure. Journal of Cellular and Molecular Medicine.

[CR20] Nguyen, N., Jennen, D., & Kleinjans, J. (2022). Omics technologies to understand drug toxicity mechanisms. *Drug Discovery Today*, 103348. 10.1016/j.drudis.2022.103348 36089240

[CR21] Olesti E, González-Ruiz V, Wilks MF, Boccard J, Rudaz S (2021). Approaches in metabolomics for regulatory toxicology applications. The Analyst.

[CR22] Rashmi D, Zanan R, John S, Khandagale K, Nadaf A (2018). γ-aminobutyric acid (GABA): Biosynthesis, role, commercial production, and applications. Studies in Natural Products Chemistry.

[CR23] Ryu HM, Kim YJ, Oh EJ, Oh SH, Choi JY, Cho JH, Kim CD, Park SH, Kim YL (2016). Hypoxanthine induces cholesterol accumulation and incites atherosclerosis in apolipoprotein E-deficient mice and cells. Journal of Cellular and Molecular Medicine.

[CR24] Schenkl C, Heyne E, Doenst T, Schulze PC, Nguyen TD (2023). Targeting mitochondrial metabolism to save the failing heart. Life.

[CR25] Sun H, Olson KC, Gao C, Prosdocimo DA, Zhou M, Wang Z, Jeyaraj D, Youn JY, Ren S, Liu Y (2016). Catabolic defect of branched-chain amino acids promotes heart failure. Circulation.

[CR26] Wajid S, Menaka M, Ahmed F, Samreen S (2019). A literature review on oral hypoglycemic drugs–mechanistic aspects. Asian Journal of Pharmaceutical and Clinical Research.

[CR27] Wei X, Shi X, Kim S, Zhang L, Patrick JS, Binkley J, McClain C, Zhang X (2012). Data preprocessing method for liquid chromatography–mass spectrometry based metabolomics. Analytical Chemistry.

[CR28] Wei Z, Zhao J, Niebler J, Hao JJ, Merrick BA, Xia M (2020). Quantitative proteomic profiling of mitochondrial toxicants in a human cardiomyocyte cell line. Frontiers in Genetics.

[CR29] Xiong Y, Jiang L, Li T (2022). Aberrant branched-chain amino acid catabolism in cardiovascular diseases. Frontiers in Cardiovascular Medicine.

[CR30] Ye Z, Wang S, Zhang C, Zhao Y (2020). Coordinated modulation of energy metabolism and inflammation by branched-chain amino acids and fatty acids. Frontiers in Endocrinology.

[CR31] Yu L, Li H, Zhang C, Zhang Q, Guo J, Li J, Yuan H, Li L, Carmichael P, Peng S (2020). Integrating in vitro testing and physiologically-based pharmacokinetic (PBPK) modelling for chemical liver toxicity assessment—A case study of troglitazone. Environmental Toxicology and Pharmacology.

