# Supplementary material for: Toxicometabolomics-based cardiotoxicity evaluation of Thiazolidinedione exposure in human-derived cardiomyocytes
Source: Metabolomics. 2024 Feb 23;20(2):24. doi: 10.1007/s11306-024-02097-z (PMC10891199; doi:10.1007/s11306-024-02097-z)
Supplement: Supplementary file 1 — Supplementary Material 1 [file 11306_2024_2097_MOESM1_ESM.docx]

Toxicometabolomics-Based Cardiotoxicity Evaluation of Thiazolidinedione Exposure in Human-Derived Cardiomyocytes

Abdullah Al Sultan^1,2^, Zahra Rattray^1^ & Nicholas J. W. Rattray^1,3^*

^1^Strathclyde Institute of Pharmacy and Biomedical Sciences, University of Strathclyde, 161 Cathedral Street, Glasgow, UK, G4 0RE

^2^Faculty of Pharmacy, Kuwait University, Safat, 13110, Kuwait

^3^Strathclyde Centre for Molecular Bioscience, University of Strathclyde

Corresponding Author [*nicholas.rattray@strath.ac.uk](mailto:*nicholas.rattray@strath.ac.uk)

**Supplementary File**

**This file is divided into two sections: supplementary data and supplementary figures.**

# Supplementary Data

## *In Vitro* Characterisation of TZD Cytotoxicity against AC16

### Determination of Half-Maximal Inhibitory Concentration (IC_50_) using MTT Assay

The effect of TZD agents on cardiomyocyte proliferation was measured using an MTT (3-(4,5-dimethylthiazol-2-yl)-2,5-diphenyltetrazolium bromide) Assay Kit (Cat. No. V13154; Thermo Fisher, Eugene, Oregon, USA). AC16 cells were seeded in 96-well microplates (Cat. No. 655180; Greiner Bio-One, Gillingham, Dorset, UK) (1 × 10^4^ cells/well) and exposed to increasing concentrations of the TZD agent (0.01, 0.1, 0.5, 1, 5, 10, and 20 μM) for 24 h. Following the incubation period, the MTT solution (5 mg/mL) was added to each well, the supernatants were removed and 50 μL of DMSO was used to dissolve the yielded formazan precipitate. A plate reader (GloMax Explorer Multimode Microplate Reader; Promega, Madison, WI, USA) was used to measure formazan absorbance at 560 nm.

Cell viability was expressed as a percentage and calculated as follows:

(A-treatment – A-blank)/(A-control – A-blank) × 100%,

where A = absorbance.

The half maximal inhibitory concentration (IC_50_) value was determined from a three-parameter nonlinear regression curve fitted to TZD concentration and the obtained absorbance values using GraphPad Prism 9 software (San Diego, CA, USA).

### Measurement of Adenosine Triphosphate Production

The CellTiter-Glo Luminescent Assay (Part No. G7570; Promega, Madison, WI, USA) was used to determine the level of cellular metabolism by measuring adenosine triphosphate (ATP) levels. Cells were seeded in white 96-well microplates (1 × 10^4^ cells/well) and incubated for 24 h. The cells were then exposed to increasing concentrations of PPAR-γ agonist (1, 5, 10, 50 and 100 μM) and incubated for a further 24 h. Subsequently, 100 μL of the CellTiter-Glo reagent was added to each well and the plates were agitated for 1 min in a shaking incubator. A luminometer (GloMax Explorer Multimode Microplate Reader) was then used to measure the luminescence.

## Toxicometabolomics Pipeline Methodology

### Micro-flow LC–MS-based Metabolomic Data Acquisition

Metabolite extracts of the AC16 cell biomass and corresponding culture media were randomised and subsequently analysed by high-performance liquid chromatography-electrospray ionisation quadrupole orbitrap mass spectrometry (HPLC-ESI-HRMS) using a Thermo Scientific™ Vanquish™ binary LC system coupled to a Thermo Scientific™ Orbitrap Exploris™ 240 mass spectrometer. The LC separations were carried out on an Accucore C18 HPLC column (2.6 μm, 100 mm × 2.1 mm I.D.; Thermo Fisher) at 40°C and operated at a flow rate of 400 μl/min. A 5 μl sample injection was used with an elution gradient consisting of water (eluent A) and acetonitrile (eluent B), each containing 0.1% formic acid.

The mass spectrometer (Orbitrap Exploris™ 240) was equipped with a heated electrospray ion source (HESI-II). The HESI-II was operated in positive (+ESI) mode and set as follows: spray voltage 3700 V; sheath gas flow rate: 40 (arbitrary units); auxiliary gas flow rate: 10 (arbitrary units); sweep gas flow rate: 1 (arbitrary units); ion transfer tube temperature: 300°C; and vaporiser temperature: 280°C. The duty cycle consisted of a full MS scan with an MS1 resolution of 60,000 and then 5 subsequent data dependent acquisition scans using 30,000 resolution and an RF lens of 70% in an m/z scan range of 70–1050. XCalibur ™ 4.2 software (Thermo Fisher Scientific) was used for data acquisition.

### Data Processing Using Compound Discoverer 3.2

The acquired LC-MS data were processed using Compound Discoverer 3.2 software (Thermo Fisher, San Jose, CA, USA). Briefly, raw data files were aligned with adaptive curve settings with 3 ppm mass tolerance and a 0.3 min retention time shift. Unknown compounds were detected with 3 ppm tolerance, 30% relative intensity tolerance and 20,000 minimum detectable peak intensity and subsequently grouped by mass tolerance and retention time tolerance by 3 ppm mass and 0.2 min, respectively. Subsequently, the normalisation procedure took place, at which peak areas across all the samples were normalised to the total area of the corresponding samples. Features identified in the processed raw data of mass spectral peaks within a 5-ppm mass error were searched against the mzCloud spectral library and ChemSpider™ databases. Databases selected by ChemSpider were the Human Metabolome Database (HMDB), BioCyc, Chemical Entities of Biological Interest (ChEBI), Kyoto Encyclopaedia of Genes and Genomes (KEGG), Taneisa Grier, Toxin, Toxin-Target Database, WikiPathways and xPharm. All data reported align to MSI Level 2 identification.

# Supplementary Figures


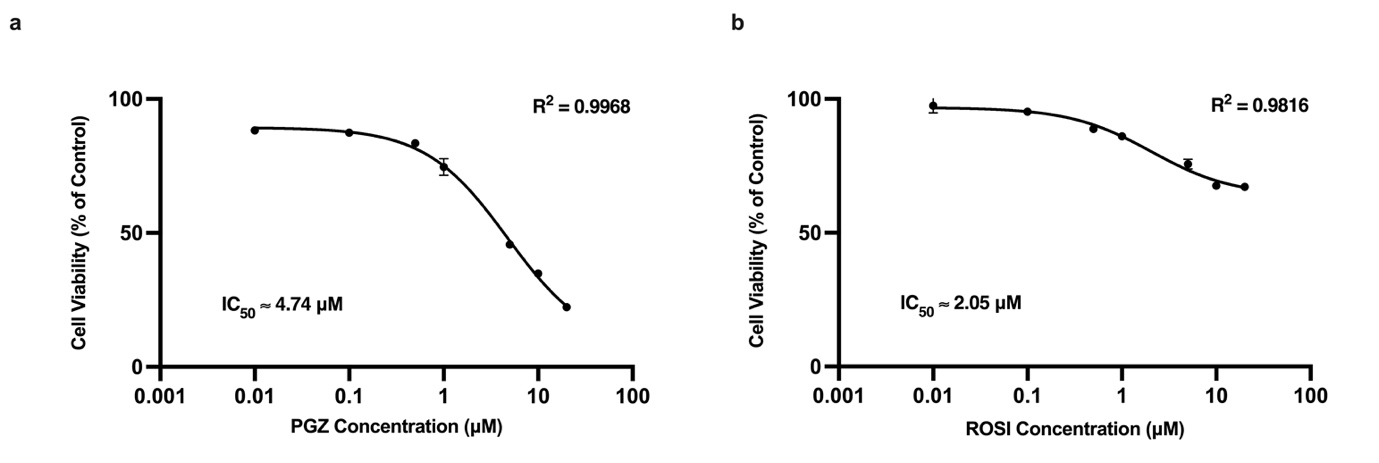


**Figure S1. Cytotoxic effects of TZDs on AC16 cells.** Dose-response modelling and corresponding IC_50_ values for PGZ (a) and ROSI exposure (b) in AC16 cells. In each experiment, cells were pre-cultured for 24 h in 96-well plates (1 × 10^4^ cells/well) and incubated with increasing concentrations of either PGZ or ROSI for another 24 h. Each point is the average of four independent experiments (each in quadruplicate), with standard deviation indicated by error bars.

TZDs: thiazolidinediones; IC_50_: half maximal inhibitory concentration; PGZ: pioglitazone; ROSI: rosiglitazone.


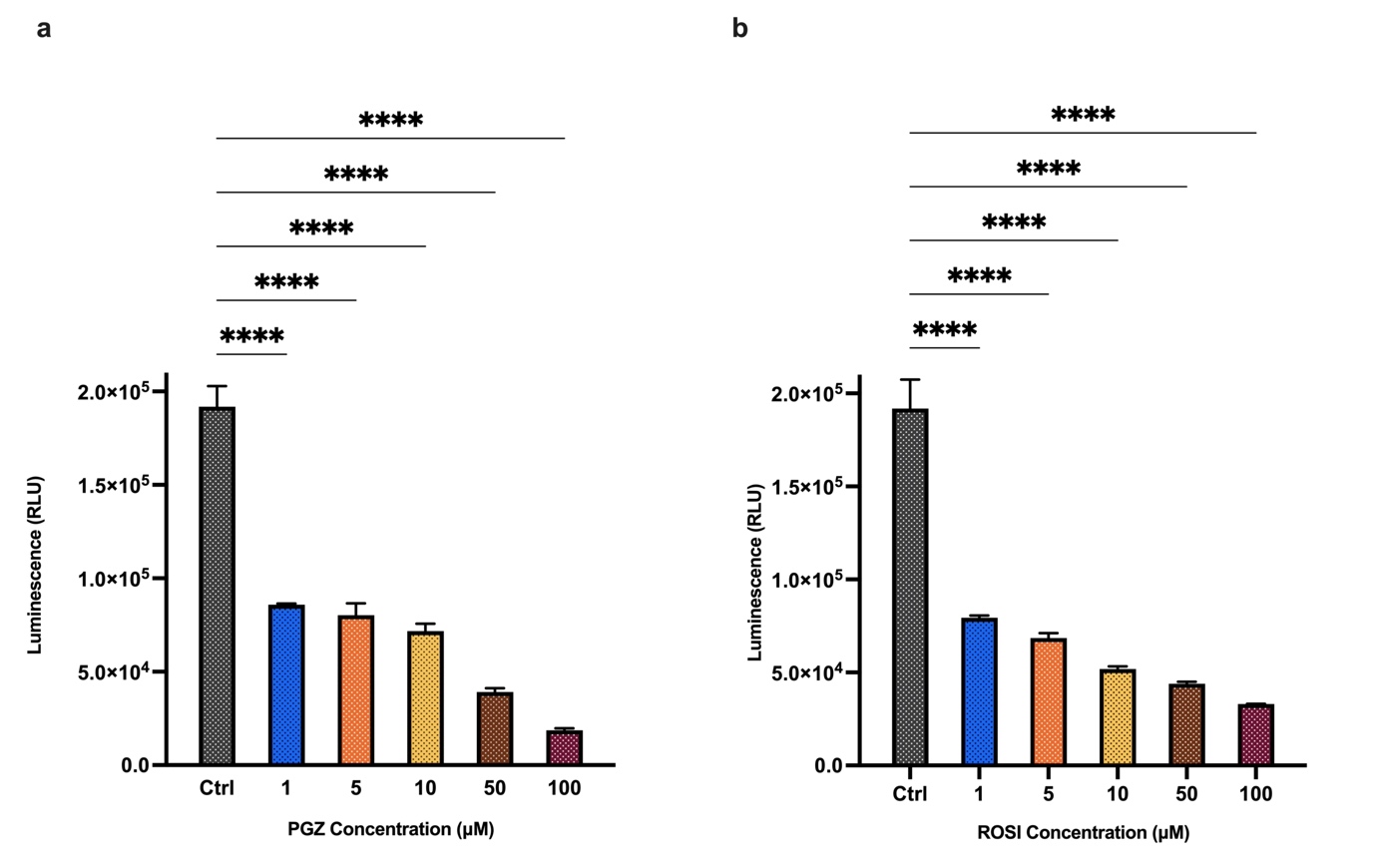


**Figure S2. The effect of the TZDs on mitochondrial ATP production.** The effect of the TZDs on mitochondrial ATP production and quantifying cellular ATP production levels of AC16 cells after 24 h of exposure to increasing concentrations of either PGZ (a) or ROSI (b) using the CellTiter-Glo luminescent assay are shown. The data are from three independent experiments, each performed in triplicate, and expressed as mean ± SD. Statistical significance was determined using one-way ANOVA and Dunnett's multiple-comparisons test (the control vs. each concentration of TZD).

**p* < 0.05, ***p* < 0.01, ****p* < 0.001 and *****p* < 0.0001

TZD: thiazolidinedione; PGZ: pioglitazone; ROSI: rosiglitazone; ATP: adenosine triphosphate


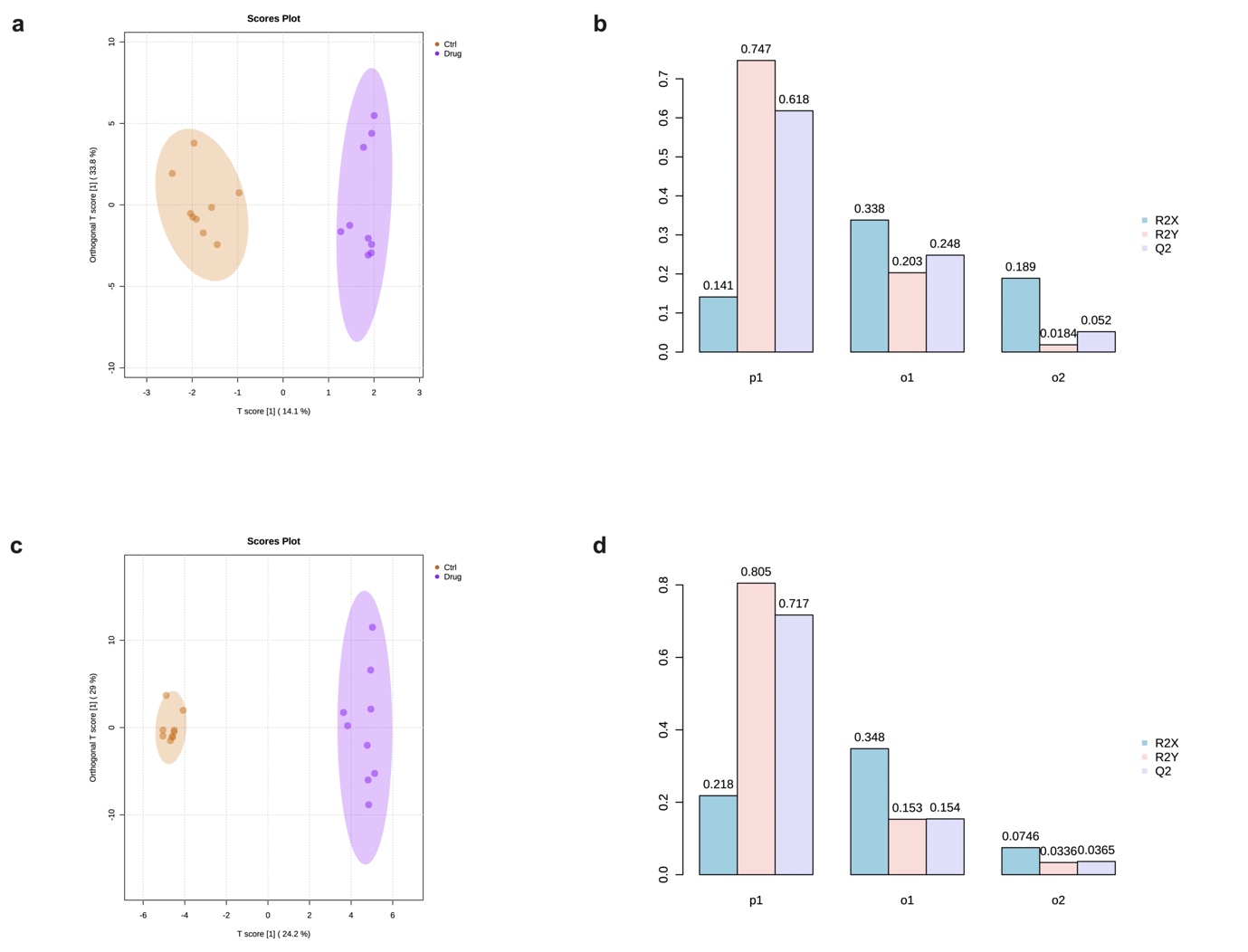


**Figure S3. Multivariate statistical analysis of the acquired LC–MS data.** OPLS-DA plots showing the separation between the (a) PGZ-treated vs. control groups and the (c) ROSI-treated vs. control groups. (b) and (d) show the cross-validated cumulative modelled variation R^2^X, R^2^Y and Q_2_ coefficients of the predictive loading (p1) and orthogonal (o1, o2) components applied on OPLS-DA data. The supervised OPLS-DA model illustrated in (a) yielded satisfactory fitness and a high predictive ability value (*R*^2^ = 0.747 and *Q*^2^ = 0.618, respectively), while the OPLS-DA score plot of the metabolite profiling data in (c) demonstrated a similar clustering panel between the experimental groups, exhibiting satisfactory fitness and predictive ability values (*R*^2^ = 0.805 and *Q*^2^ = 0.717, respectively).

PGZ: pioglitazone; ROSI: rosiglitazone; OPLS-DA: orthogonal partial least squares-discriminant analysis.

**
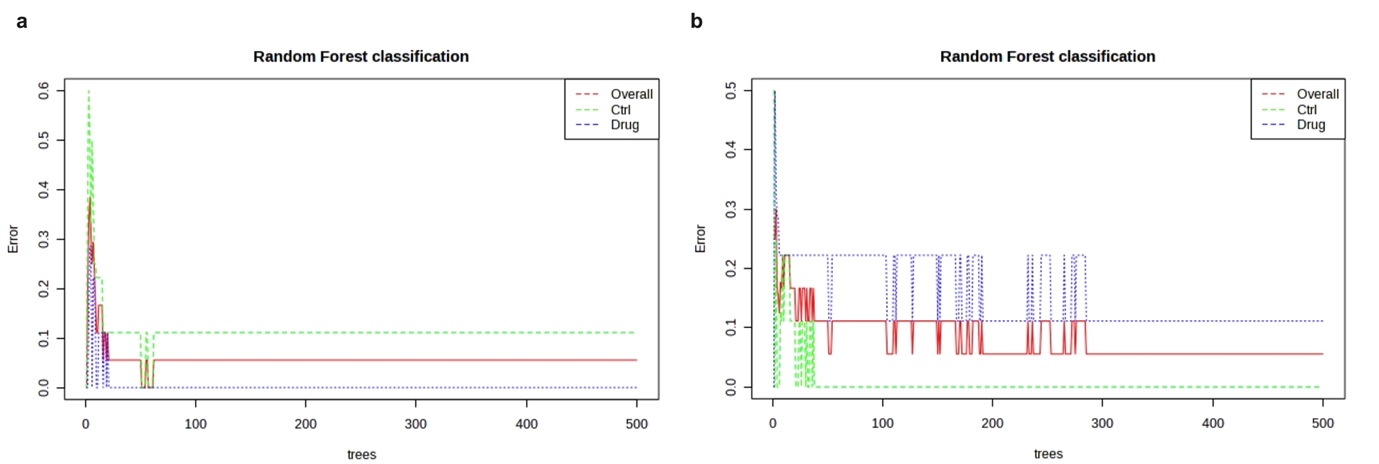
**

**Figure S4. Random forest classification model.** (a) and (b) represent the random forest classification model of the PGZ and ROSI experiments, respectively, illustrating the cumulative error rates measured for each experimental group using the machine learning approach.

PGZ: pioglitazone; ROSI: rosiglitazone


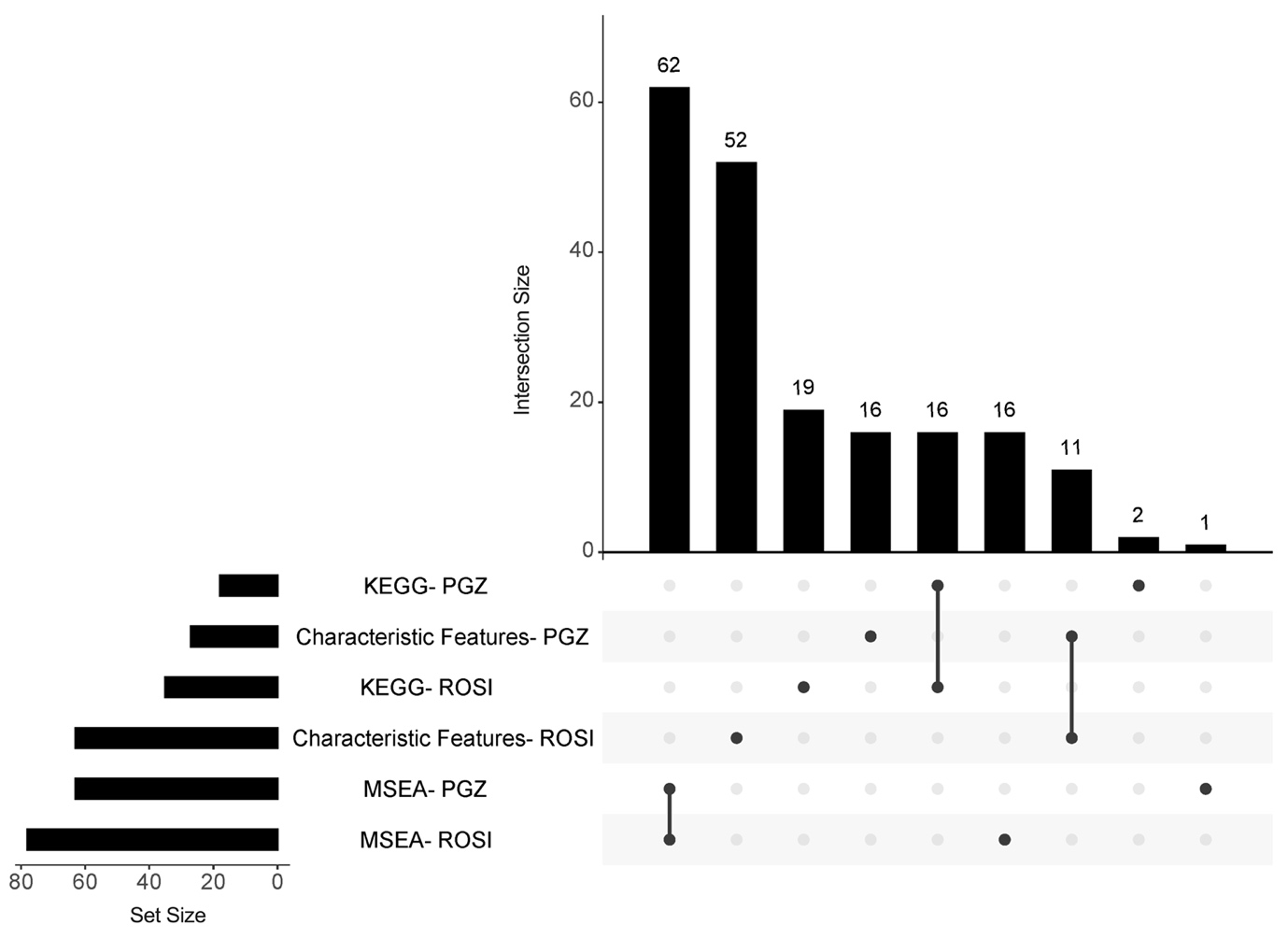


**Figure S5. UpSet plot illustrating the overlapping and specific characteristic features, MSEA and pathway analysis findings for the two experiments.** The *x*-axis (set size) represents the size of each set, while the intersection size indicates the number of each set’s measures that are common between sets. The black points denote the intersections, while the grey points represent no intersections.

PGZ: pioglitazone; ROSI: rosiglitazone; MSEA: metabolite set enrichment analysis; KEGG: Kyoto Encyclopedia of Genes and Genomes.
